# The *Moonwalker* Mouse: New Insights into TRPC3 Function, Cerebellar Development, and Ataxia

**DOI:** 10.1007/s12311-014-0564-5

**Published:** 2014-05-06

**Authors:** Esther B. E. Becker

**Affiliations:** MRC Functional Genomics Unit, Department of Physiology, Anatomy and Genetics, University of Oxford, South Parks Road, Oxford, OX1 3PT UK

**Keywords:** Cerebellum, Ataxia, Purkinje cell, Unipolar brush cell, Mouse model, TRPC3 channel

## Abstract

The *Moonwalker* (*Mwk*) mouse is a recent model of dominantly inherited cerebellar ataxia. The motor phenotype of the *Mwk* mouse is due to a gain-of-function mutation in the gene encoding the cation-permeable transient receptor potential channel (TRPC3). This mutation converts a threonine into an alanine in the highly conserved cytoplasmic S4–S5 linker of the channel, affecting channel gating. TRPC3 is highly expressed in cerebellar Purkinje cells and type II unipolar brush cells that both degenerate in the *Mwk* mouse. Studies of the *Mwk* mouse have provided new insights into the role of TRPC3 in cerebellar development and disease, which could not have been predicted from the *Trpc3* knockout phenotype. Here, the genetic, behavioral, histological, and functional characterization of the *Mwk* mouse is reviewed. Moreover, the relationship of the *Mwk* mutant to other cerebellar mouse models and its relevance as a model for cerebellar ataxia are discussed.

## Introduction

The cerebellar ataxias are a highly heterogeneous group of neurological disorders that impinge on the cerebellum and its afferent and efferent connections. Patients affected by cerebellar ataxia suffer from poor coordination of movements, imbalance, and a wide-based unsteady gait. Patients also exhibit various combinations of oculomotor deficits, dysarthria, and kinetic tremor. Moreover, patients often present with additional extra-cerebellar signs including marked cognitive dysfunction. Ataxias are rare diseases with a varying prevalence between 1 and 40:100,000 [1, 2]. The ataxias are broadly classified into hereditary ataxias, nonhereditary degenerative ataxias, and acquired ataxias [3, 4]. The hereditary ataxias comprise a growing list of genetically diverse disorders with over 43 genetic loci known to cause the autosomal dominant ataxias and altogether more than 100 genes that primarily cause ataxia when mutated [1–3]. However, there are still several clinically distinct forms of hereditary ataxias for which the disease-causing mutations have not been identified, and additional genes underlying cerebellar ataxia remain to be discovered. The vast number of genes implicated in ataxia suggests that multiple pathogenic pathways can induce cerebellar dysfunction and atrophy. Indeed, the pathogenesis of cerebellar ataxia appears to be highly heterogeneous, and a multitude of potential disease mechanisms have been proposed. Many of these are not ataxia-specific but common disease features of neurodegenerative disorders in general, such as oxidative stress, protein aggregation, and transcriptional dysregulation [3, 5]. The challenge remains to better understand the specific disease-causing mechanisms underlying the complex cerebellar ataxias and to identify common pathological pathways that could be targeted therapeutically.

Animal models provide invaluable insights into the normal function of the cerebellum and into the pathogenic mechanisms contributing to cerebellar ataxia. A large number of mouse mutants exhibiting a cerebellar ataxia phenotype have been reported (reviewed in [6, 7]). Historically, the first ataxic mouse models that were intensively investigated were naturally occurring mutants, including the *Lurcher* (gain-of-function mutation in *Grid2*), *staggerer* (*Rora* deletion), and *Weaver* mouse (gain-of-function mutation in *Girk2*). For some of these spontaneous mutants, a direct clinical relevance was demonstrated only years after their initial discovery. For example, the mutation in the *staggerer* mouse was first observed at the Jackson Laboratory in 1955 and later identified as a null allele of the retinoid acid-related orphan nuclear receptor-alpha (RORα) [8]. More than 40 years after the initial description of the *staggerer* mouse, expression profiling studies suggested a mechanistic link between RORα and spinocerebellar ataxia (SCA) type 1 (SCA1) [9, 10]. Subsequently, RORα was shown to physically interact in a regulatory transcriptional complex with ATXN1, the mutated protein in SCA1 [11]. Similarly, it took 59 years after the initial discovery of the *Lurcher* mutant, until the identification of the first human mutations in *GRID2* in patients with cerebellar ataxia [12, 13]. These findings underscore the often tedious search for human genes implicated in the rare cerebellar ataxias, but also the power of ataxic mouse mutants in dissecting the disease-relevant molecular mechanisms of cerebellar dysfunction.

In addition to the spontaneous mutants, many transgenic, knockout, and knockin mouse lines have been engineered for the human ataxias with varying degrees of success. Some of these mutants, particularly the generated mouse models of the trinucleotide-repeat SCAs, phenocopy the human condition reasonably well and have been valuable tools in analyzing the underlying molecular pathogenesis and discovering potential new therapeutics for cerebellar ataxia [14]. Genome-wide random mouse mutagenesis represents a powerful complementary approach to gene knockouts and transgenics for the discovery of novel genes and allelic gene series with functional implications for the central nervous system [15–17]. This phenotype-driven approach has been particularly successful in identifying cerebellar mutants, partly due to the relatively easy detection of motor phenotypes in the mouse. As part of a large *N*-ethyl-*N*-nitrosurea (ENU) mutagenesis screen to identify new models of human cerebellar ataxia, we identified the dominantly inherited *Moonwalker* (*Mwk*) mouse [18]. Here, I review the genetic and functional characterization of the *Mwk* mouse, which has provided new insights into the role of the transient receptor potential (TRP) channel TRPC3 in cerebellar development and disease [18, 19]. I also examine the relationship of the *Mwk* mutant to other mouse models of cerebellar ataxia and its relevance for the human disorder.

## The *Mwk* Mouse: Gain-of-Function Mutation in the *Trpc3* Gene

**Fig. 1:**
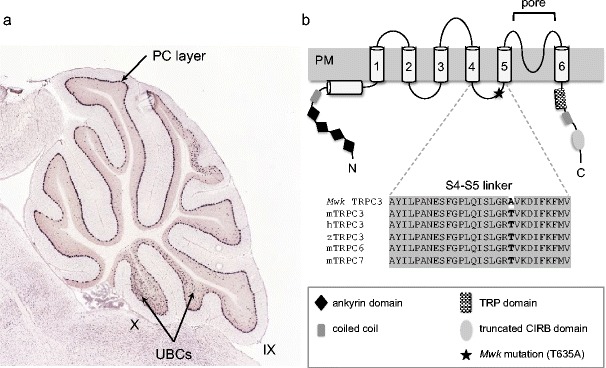
**a** TRPC3 mRNA expression in the adult cerebellum. TRPC3 is predominantly expressed in the Purkinje cell (*PC*) layer. In addition, intense labeling of unipolar brush cells (UBCs) in lobules IX and X is apparent. In situ hybridization image is taken from the Allen Mouse Brain Atlas, available from http://mouse.brain-map.org. **b** Domain structure and transmembrane topology of TRPC3. TRPC3 contains four ankyrin-like repeats at the cytoplasmic N terminus, an N-terminal coiled coil region and a hydrophobic region, which does not span the plasma membrane (*PM*), six transmembrane regions, a pore region, a characteristic TRP domain in the cytoplasmic C-terminal region, followed by a coiled coil and truncated calmodulin and IP_3_R1-binding domain (*CIRB*). The localization of the *Mwk* mutation (T635A) in the cytoplasmic S4–S5 linker region is indicated. Multiple sequence alignment between mouse mTRPC3, human hTRPC3, and the related mouse mTRPC6 and mTRPC7 S4–S5 linker shows a high degree of sequence conservation

*Mwk* mice harbor a single non-synonymous point mutation in exon 7 of the *Trpc3* gene located on chromosome 3 [18]. This gene encodes the TRPC3 channel, a member of the transient receptor potential (TRP) family of ion channels. The TRP superfamily constitutes one of the largest ion channel families and is involved in numerous physiological functions [20, 21]. The mammalian TRP channel proteins form six-transmembrane cation-permeable channels that are grouped into six subfamilies including the canonical TRPC family (TRPC 1–7). TRPC channels are widely expressed in different mouse tissues including the nervous system, where they function as non-selective calcium entry channels with distinct modes of activation [22, 23]. Microarray analysis of more than 80 different mouse cell lines and tissues shows that TRPC3 is highly expressed in the nervous system, particularly in the cerebellum (BioGPS gene portal) (http://Biogps.org). Notably, it is the alternatively spliced short isoform TRPC3c that is preferentially expressed in the cerebellum [24]. This TRPC3 isoform lacks part of the CIRB domain (see Fig. 1b), which is known to modulate TRPC channel activity via competitive binding of the inositol triphosphate (IP_3_) receptor (IP_3_R) and calmodulin (CaM), resulting in a channel with enhanced efficacy due to increased channel-opening frequency [24]. Within the cerebellum, TRPC3 is most highly expressed on the soma as well as the dendrites of Purkinje cells [25, 26] (Fig. 1a). In fact, TRPC3 was shown to be the most abundant TRP channel in Purkinje cells [25]. In addition to Purkinje cells, TRPC3 was recently found to be selectively expressed in type II unipolar brush cells (UBCs), which are excitatory glutamatergic interneurons in the cerebellum (Fig. 1a) [19]. Using a *Trpc3* knockout mouse model, Hartmann et al. have demonstrated that TRPC3 mediates metabotropic glutamate receptor subtype 1 (mGluR1)-dependent synaptic transmission in Purkinje cells [25]. Similarly, activation of group I mGluR gates TRPC3 channels in type II UBCs [19]. Moreover, TRPC3 was recently suggested to be essential for the induction of long-term depression (LTD) in cerebellar Purkinje cells [27]. However, how loss of synaptic function in the *Trpc3* mouse mutants affects LTD remains to be determined.

The *Mwk* mutation results in a threonine-to-alanine amino acid change (T635A; RefSeq# NP_062383)^1^ in the highly conserved cytoplasmic S4–S5 linker region of the TRPC3 protein (Fig. 1b). The *Mwk* mutation does not alter the normal expression pattern of TRPC3. However, electrophysiological recordings from *Mwk* Purkinje cells revealed that mutant TRPC3 exhibits altered gating properties that promote channel opening under conditions of low mGluR1 activation [18]. Consistently, *Mwk* but not wild-type TRPC3 promotes increased calcium signaling and cell death upon overexpression in neuronal cell lines [18] (unpublished observations). The molecular mechanisms of how the *Mwk* mutation leads to abnormal TRPC3 channel gating remain to be fully elucidated. One possible mechanism might be the loss of an inhibitory phosphorylation by protein kinase C γ (PKCγ). PKCγ has been shown to inhibit TRPC3 channel activity in overexpression experiments in heterologous cell lines [28–31]. Indeed, threonine 635, which is mutated in the *Mwk* mouse, has been shown to be phosphorylated by PKCγ in an in vitro kinase assay [18]. However, this inhibitory phosphorylation event has not yet been confirmed in vivo. Moreover, Nelson and Glitsch have argued that native TRPC3-dependent currents elicited in cerebellar Purkinje cells are unlikely to be targets of conventional PKC or PKG kinases in contrast to the findings observed in overexpression experiments [32]. Alternatively, the *Mwk* mutation in the S4–S5 linker might disturb intramolecular interactions within TRPC3 that are important for the gating mechanism of the channel. This mechanism might be similar to the established electromechanical coupling in the structurally related voltage-gated potassium channels, in which the S4–S5 linker is critical for gating [33]. Recently, the S4–S5 linker in TRPC4 and TRPC5 channels was identified as critical constituent of TRPC4/5 channel gating [34], suggesting a similar key function for the S4–S5 linker in TRPC3. Future structural studies on TRPC3 and related TRP channels will undoubtedly shed more light on the intramolecular interactions that are crucial for TRPC3 channel activation and function.

## The *Mwk* Phenotype

**Fig. 2:**
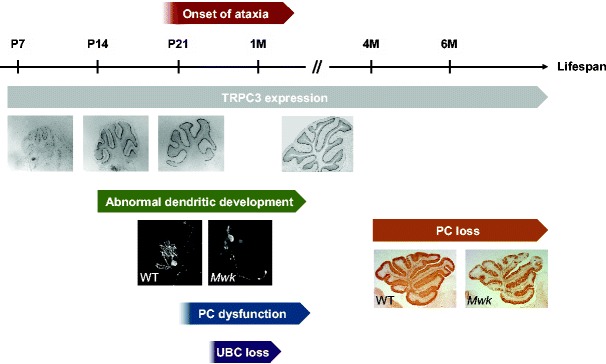
Progression of the *Mwk* phenotype from the early postnatal period until adulthood. TRPC3 expression starts early postnatally and remains high during adulthood. In situ hybridization images show *Trpc3* expression at P8, P13, P18, and P56. Developing mouse sections are more laterally compared to the midline section shown for the adult. Importantly, dendritic abnormalities, Purkinje cell (*PC*) dysfunction, and overt ataxia in the *Mwk* mouse appear prior to the onset of PC loss. Calbindin-stained parasagittal sections from 6-month-old mice are shown. Most of the unipolar brush cells (*UBCs*) are lost by 1 month of age; it remains to be determined when the loss of these neurons starts. Figure modified from Becker et al. [18]. *M* months, *Mwk Moonwalker* mouse, *P* postnatal day, *PC* Purkinje cell, *UBC* unipolar brush cell, *WT* wild-type

From early postnatal days, *Mwk* mouse mutants display growth retardation and remain about 60 % the size of their wild-type littermates throughout life. Homozygous mutants (*Mwk*/*Mwk*) are embryonically lethal. Heterozygous *Mwk* mouse mutants display motor and coordination defects from about 3 weeks of age [18] (Fig. 2). Specifically, *Mwk* mice exhibit retropulsion and a wider and “shuffling” gait compared to wild-type littermates. *Mwk* mice are also severely impaired in their ability to maintain their balance in the static rod test. Following their initial behavioral characterization, *Mwk* mice are being extensively characterized as part of the EuroPhenome project [35]. In this large-scale phenotyping effort, *Mwk* mutants were found to display significant hyperactivity, tail elevation, and hind limb grasping behavior, indicative of extra-cerebellar signs. Many other behavioral tests in the EuroPhenome pipelines are still ongoing.

Notably, the *Trpc3* knockout mice also exhibit an ataxic phenotype, albeit much milder than the *Mwk* mice [25]. The fact that both loss and gain of TRPC3 function lead to cerebellar ataxia has been previously attributed as “conflicting” [36]. However, it is commonly observed in other ataxic mouse mutants as well as human ataxia patients that both decreased and increased ion channel activities can lead to Purkinje cell dysfunction and consequently cerebellar ataxia [6, 37]. This established concept that functionally different mutations in the same ion channel gene can cause the same disease phenotype highlights the importance of the associated biological mechanisms such as maintenance of calcium homeostasis for proper Purkinje cell function.

Histopathological analysis of the *Mwk* brain revealed a slow but progressive loss of Purkinje cells starting at 4 months of age [18] (Fig. 2). Purkinje cell degeneration is particularly pronounced in the lateral hemispheres of the cerebellum. Moreover, type II UBCs are dramatically reduced by 1 month of age [19]. The mode of cell death in the *Mwk* cerebellum remains unclear. No evidence of DNA fragmentation of activation of pro-apoptotic signaling molecules was found, suggesting a cell death mechanism other than apoptosis. Given the underlying gain-of-function mutation in TRPC3 and observed increase in calcium signaling, cell death is likely to occur due to calcium overload. This mechanism might also explain the fact that type II UBCs are lost much earlier compared to Purkinje cells in the *Mwk* cerebellum. Purkinje cells have an extensive calcium-buffering capacity due to the presence of high concentrations of calcium-binding proteins including parvalbumin and calbindin. The latter has been estimated to comprise more than 15 % of total protein in Purkinje cells [38]. Hence, the ability to handle an excessive influx of calcium is likely to be much more rapidly exceeded in UBCs compared to Purkinje cells. It would be interesting to further dissect the relative contribution of UBC loss and Purkinje cell dysfunction with regard to the ataxic *Mwk* phenotype in a conditional mouse mutant. Type II UBCs are predominantly found in the vestibulocerebellum, suggesting that their loss in the *Mwk* mouse might contribute to the profound balance impairment of the mutant.

## Abnormal Cerebellar Development in the *Mwk* Mouse

Significant cerebellar TRPC3 expression starts within the second postnatal week and peaks at 3 weeks postnatally [18] (see also Cerebellar Development Transcriptome Database) (http://www.cdtdb.neuroinf.jp) (Fig. 2), suggesting a role for this non-selective cation channel during Purkinje cell development. Interestingly, *Mwk* mice exhibit ataxic behavior long before loss of Purkinje cells is observed (Fig. 2), indicating that the ataxic phenotype is caused by the dysfunction rather than the loss of Purkinje cells. Indeed, electrophysiological alterations are evident in the *Mwk* Purkinje cells as early as 3 weeks of age [18, 19]. Cell recordings showed that only a small fraction of *Mwk* Purkinje cells are spontaneously active and suggested that *Mwk* Purkinje cells are generally depolarized [19]. Consistent with these observations, *Mwk* Purkinje cells responded differently to stimulation of mGluR1, with either no inward current or an inward current with significantly smaller spikes at a higher frequency [18]. Together, these findings suggest that Purkinje cells in the developing *Mwk* cerebellum are profoundly impaired in their intrinsic and evoked electrophysiological properties.

Developmental abnormalities in the *Mwk* cerebellum are also evident in the altered dendritic morphology of the mutant Purkinje cells. The postnatal increase in TRPC3 expression coincides with the most intensive phase of dendritic arborization of Purkinje cells, suggesting a role for TRPC3 in this process. Consistent with this idea, *Mwk* Purkinje cell dendritic arbors were found to be significantly less elaborate compared to wild-type Purkinje cells [18] (Fig. 2). This phenomenon was particularly apparent in organotypic slice cultures of the developing cerebellum, where *Mwk* Purkinje cells exhibit a profoundly reduced dendritic arbor. This dendritic phenotype in the *Mwk* mice that have increased cerebellar calcium signaling is consistent with other studies showing reduced Purkinje cell dendritic growth upon activation of mGluR1 [39, 40] and PKC [41–43]. Recently, voltage-gated calcium channels (VGCCs) were implicated in the regulation of Purkinje cell dendritic growth after chronic mGluR1 or PKC activation [44]. Interestingly, genetic knockout of *Trpc3* or pharmacological inhibition of TRPC3 did not alter Purkinje cell dendritic arborization [44]. Together, these findings suggest that it is the excessive calcium influx during the critical period of dendritic development in *Mwk* mice that causes inhibition of dendritic growth. The inhibition of Purkinje cell dendritic arborization by increased calcium signaling through activation of mGluR1, TRPC3, and PKC might be a powerful negative feedback mechanism to limit the size of the dendritic arbor after the establishment of a sufficient number of granule cell parallel fiber contacts. This model is consistent with earlier findings that electric activity controls Purkinje cell dendritogenesis [45]. In further support of this, Purkinje cells of the *Lurcher* mouse with a gain-of-function mutation in the δ2 glutamate receptor (GluD2), which changes the receptor into a leaky membrane channel resulting in chronic depolarization, also exhibit profoundly impaired dendritic growth [46].

An extensive literature supports a role for calcium signaling in the dendritic morphogenesis of neurons including the local regulation of dendritic branch dynamics as well as the global control of gene transcription (reviewed in [47, 48]). However, surprisingly, little is known about the specific calcium-activated signaling mechanisms that regulate Purkinje cell dendritic arborization downstream of mGluR1, TRPC3, and VGCCs. PKCs have emerged as one of the kinases that regulate Purkinje cell dendritic arborization in response to increased calcium influx (reviewed in [43, 49]). It appears that both PKC isoforms, PKCα and PKCγ, contribute to Purkinje cell dendritic development, depending on the strength of the stimulus. Furthermore, calmodulin-dependent kinases (CaMKs) have been implicated in Purkinje cell dendritic growth. Pharmacological block of CaMKs has been reported to reduce the number of Purkinje cell primary dendrites in cerebellar cultures [50]. This effect was only observed between 5 and 15 days in vitro but not later, suggesting that CamKs might be involved in the early phases of Purkinje cell dendritogenesis and before a critical function of TRPC3 in this process. The specific substrates of PKCs and CamKs that regulate Purkinje cell dendritic development remain largely unknown. The only reported downstream effector of CamKII signaling in Purkinje cells controlling local dendritic branching is the microtubule destabilizer stathmin, which is phosphorylated by CamKII upon activation of VGCCs and mGluR1 [51]. Thus, further studies are needed to dissect the molecular mechanisms that control the calcium-dependent Purkinje cell dendritic growth during development. The *Mwk* mouse provides an excellent model system to study the transcription-dependent and transcription-independent effector pathways that regulate the developmental dendritic arborization of Purkinje cells. Efforts are underway to identify the genes and pathways that mediate Purkinje cell dendritic growth upon TRPC3 activation. Importantly, as Purkinje cells constitute significantly less than 1 % of the total cerebellar cell population [52], Purkinje cell-specific transcriptional and biochemical profiling requires enriched cell preparations obtained by cell sorting or laser microdissection [53, 54].

## The Relevance of TRPC3 Signaling for Cerebellar Ataxia

**Fig. 3:**
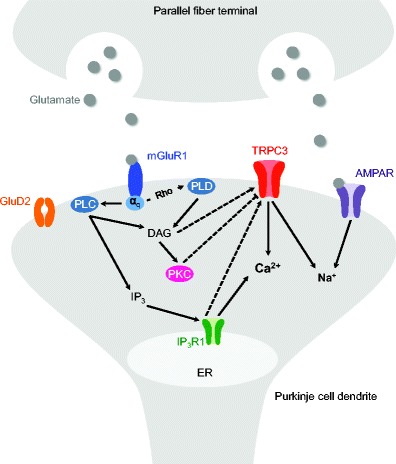
mGluR1-TRPC3 signaling at parallel fiber-Purkinje cell synapses. TRPC3 is central to the glutamate-triggered pathway that is vital for Purkinje cell function and, when disrupted, results in cerebellar ataxia. Please see main text for details. *AMPAR* AMPA receptor, *GluD2* δ2 glutamate receptor, *IP*_*3*_ *R1* inositol triphosphate receptor type 1, *mGluR1* metabotropic glutamate receptor subtype 1, *PKC* protein kinase C, *PLC* phospholipase C, *PLD* phospholipase D, *TRPC3* transient receptor potential channel C3

Mouse knockout studies have demonstrated a key role for TRPC3 in mediating mGluR1-dependent synaptic transmission in Purkinje cells [25]. In the molecular layer, Purkinje cells receive major glutamatergic input from granule cell parallel fibers. Glutamate released from the parallel fiber presynaptic terminals binds to AMPA receptors and mGluR1 at the parallel fiber-Purkinje cell synapse (Fig. 3). Influx of sodium ions through AMPA receptor channels leads to a rapid postsynaptic depolarization, whereas mGluR1s mediate a local dendritic calcium signal and a prolonged depolarization known as slow excitatory postsynaptic current (slow EPSC) (reviewed in [55]). Glutamate binding to mGluR1 activates phospholipase C (PLC) via G protein Gα_q_ to produce IP_3_ and diacylglycerol (DAG). IP_3_ subsequently activates calcium-permeable IP_3_R1 receptor channels on the endoplasmic reticulum (ER) membrane, resulting in a characteristic local dendritic calcium response that is required for the induction of LTD [56]. The mGluR1-dependent slow EPSC is mediated by TRPC3 [25]. The exact gating of TRPC3 following of mGluR1 activation remains to be elucidated but might involve Rho GTPase-dependent activation of phospholipase D1 (PLD1) [57] as well as IP_3_R and calmodulin [58] and others (reviewed in [59, 60]). TRPC3 activity has been proposed to be negatively regulated through phosphorylation by PKCγ and perhaps other kinases [28–31, 59], but the exact nature of this inhibitory phosphorylation remains controversial [32].

Importantly, disruption of any component of the mGluR1-triggered signaling cascade results in cerebellar dysfunction and disease. Similar to the *Mwk* mouse phenotype, knockout mice deficient in the genes encoding mGluR1 [61, 62], Gα_q_ [63], IP_3_R1 [64], TRPC3 [25], and PKCγ [65] all exhibit cerebellar ataxia. Moreover, auto-antibodies against mGluR1 are associated with neoplastic as well as subacute cerebellar ataxia in human patients [66–68]. Also, deletions in the *ITPR1* gene encoding IP_3_R1 cause SCA15 [69]. Gain-of-function missense mutations in the *PRKCG* gene encoding PKCγ cause SCA14 [70]. Interestingly, SCA14-associated PKCγ mutants were shown to fail to phosphorylate TRPC3, resulting in a sustained calcium influx that might be central to the SCA14 pathogenesis [30]. Thus, the *Mwk* mouse might represent a relevant model for SCA14. Overexpression of SCA14-associated PKCγ mutations in developing Purkinje cells also disturbs their dendritic development [71], which is consistent with the *Mwk* developmental phenotype.

Recently, activation of mGluR1 was shown to also trigger gating of the postsynaptic GluD2 receptor [72]. In another study, GluD2 was found to associate with a mGluR1-TRPC3-PKCγ signaling complex and to regulate mGluR1-mediated synaptic transmission in cerebellar Purkinje neurons [73]. Interestingly, in this study, genetic loss of GluD2 was found to increase extra-synaptic mGluR1 and TRPC3 expression in the *hotfoot* mutant mice, resulting in abnormal synaptic transmission. Both loss-of-function and gain-of-function mutations in the *Grid2* gene encoding GluD2 result in defects in Purkinje cell dendritic arborization and cerebellar ataxia [6]. Recently, the first mutations in the human *GRID2* gene have been reported in patients with cerebellar ataxia [12, 13].

In addition to the direct links between disrupted glutamatergic signaling and cerebellar ataxia described above, increasing evidence points towards a role of perturbed TRPC3 signaling in other genetic forms of cerebellar ataxia. For example, the expression of TRPC3 and other genes involved in calcium homeostasis was shown to be downregulated in a mouse model of SCA1 [74]. Importantly, these expression changes occurred early and before any observed pathological changes in SCA1, suggesting that they might be a causal factor in the pathogenesis of SCA1. Consistent with these findings, mGluR1-TRPC3-mediated synaptic signaling is disrupted in homozygous *staggerer* mice, an extreme model of SCA1 [75]. Both expression of mGluR1 and TRPC3 were found to be dramatically reduced in these mice, and consequently, slow EPSCs were absent. Similarly, mGluR1-mediated synaptic transmission is completely absent in a transgenic mouse model of SCA3 [76].

Collectively, these studies underscore that TRPC3 is a central player in a glutamate-triggered signaling cascade that is vital for Purkinje cell function and, when disrupted, results in cerebellar disease in mice and men (Fig. 3). Moreover, these findings suggest that *TRPC3* itself might be a promising candidate gene for human cerebellar ataxia. Supporting this, a methylation-regulating *TRPC3* promoter polymorphism was found to be enriched in patients with idiopathic cerebellar ataxia [77]. So far, candidate screening of the *TRPC3* gene did not identify potential mutations in patients with sporadic late-onset cerebellar or episodic ataxia, suggesting that *TRPC3* mutations are unlikely to be a common cause of cerebellar ataxia [78]. However, *TRPC3* mutations might still contribute to cerebellar ataxia in specific subtypes of the disease that have not been screened yet. The increasing use of next-generation sequencing approaches for the genetic diagnosis of cerebellar ataxia is poised to fully elucidate the role that *TRPC3* mutations may play in human cerebellar ataxia [79].

## Conclusions

The gain-of-function *Mwk* mouse has provided novel insights into the function of TRPC3 in the normally developing cerebellum as well as in cerebellar ataxia. In particular, the link between TRPC3-triggered aberrant Purkinje cell development and cerebellar ataxia could not have been predicted from the *Trpc3* knockout phenotype, underscoring the power of genome-wide random mouse mutagenesis in identifying important mechanisms underlying nervous system dysfunction. Accumulating evidence from other cell- and animal-based models of cerebellar ataxia suggests that abnormal Purkinje cell development and early changes in Purkinje cell physiology commonly contribute to cerebellar ataxia, thus challenging on our view of cerebellar ataxia as a neurodegenerative disorder [11, 71, 80, 81].

Despite major progress in deciphering the genetic and molecular mechanisms of cerebellar ataxia, effective therapies for this group of neurological diseases are still lacking. One of the biggest challenges of ataxia research is the identification of drug targets and the development of novel therapeutic approaches. The *Mwk* mouse and other cerebellar mutants have highlighted the importance of synaptic transmission at the parallel fiber-Purkinje cell synapse and thus mGluR1-TRPC3 signaling in cerebellar function and disease. Indeed, abnormal mGluR1-TRPC3 signaling seems to be a unifying feature of many hereditary ataxias including the more common trinucleotide expansion disorders SCA1 and SCA3. Thus, pharmacological regulation of this pathway might be beneficial not only in one particular ataxia subtype but in a larger group of ataxias that share a common pathological mechanism. Modulating mGluR1-TRPC3 signaling in cerebellar ataxia is particularly attractive, as it would target neuronal dysfunction early on during the course of the disease. Given the importance of the TRPC3 pathway in cerebellar ataxia, the *Mwk* mouse promises to be a valuable model to test novel compounds that might be beneficial in human cerebellar ataxia. The TRPC3 channel itself might be an attractive target to modulate in cerebellar ataxia. Hopefully, more selective and potent pharmacological modulators of TRPC3 will be developed in the near future that could hold great promise as therapeutic agents for cerebellar ataxia.

## Footnotes

1 T561A according to UniprotKB #Q9QZC1
